# Cancer stemness and metastatic potential of the novel tumor cell line K3: an inner mutated cell of bone marrow-derived mesenchymal stem cells

**DOI:** 10.18632/oncotarget.17133

**Published:** 2017-04-17

**Authors:** Hui Qian, Xiaoqing Ding, Jiao Zhang, Fei Mao, Zixuan Sun, Haoyuan Jia, Lei Yin, Mei Wang, Xu Zhang, Bin Zhang, Yongmin Yan, Wei Zhu, Wenrong Xu

**Affiliations:** ^1^ Jiangsu Key Laboratory of Medical Science and Laboratory Medicine, School of Medicine, Jiangsu University, Zhenjiang, P. R. China; ^2^ Faculty of Medical Laboratory Science, Rui-Jin Hospital, Shanghai Jiao Tong University School of Medicine, Shanghai, P. R. China

**Keywords:** mesenchymal stem cell, side population cells, cancer stem cell, metastasis, epithelial-mesenchymal transition

## Abstract

Mesenchymal stem cells (MSCs) transplantation has been used for therapeutic applications in various diseases. Here we report MSCs can malignantly transform *in vivo*. The novel neoplasm was found on the tail of female rat after injection with male rat bone marrow-derived MSCs (rBM-MSCs) and the new tumor cell line, K3, was isolated from the neoplasm. The K3 cells expressed surface antigens and pluripotent genes similar to those of rBM-MSCs and presented tumor cell features. Moreover, the K3 cells contained side population cells (SP) like cancer stem cells (CSCs), which might contribute to K3 heterogeneity and tumorigenic capacity. To investigate the metastatic potential of K3 cells, we established the nude mouse models of liver and lung metastases and isolated the corresponding metastatic cell lines K3-F4 and K3-B6. Both K3-F4 and K3-B6 cell lines with higher metastatic potential acquired more mesenchymal and stemness-related features. Epithelial-mesenchymal transition is a potential mechanism of K3-F4 and K3-B6 formation.

## INTRODUCTION

Mesenchymal stem cells (MSCs) are a type of adult stem cell that possesses self-renewal and multilineage properties [1]. Researchers have revealed that MSCs promote tumor progression by potentiating tumor growth and promoting metastasis [2–4]. Furthermore, MSCs may undergo transformation and lead to cancer development or cancer growth maintenance [5]. However, research on transformed MSCs is limited, particularly on their metastatic potential and cancer stemness.

In our previous study, a tumor cell line named F6 was cloned from mutated human MSCs *in vitro*. The F6 cells maintained the self-renewal and differentiation abilities of MSCs and possessed a high capacity for tumorigenicity. Another study reported the presence of a small population of cancer stem cells (CSCs) in F6, suggesting that mutated MSCs are a source of CSCs [6, 7].

In this study, we investigated whether MSCs can malignantly transform *in vivo*. We injected bone marrow-derived MSCs (BM-MSCs) from male rats into the tails of female rats and found neoplasms on the tails of 2 out of 10 rats individually, which were identified as fibrosarcomas. Subsequently, we established a novel tumor cell line, named K3, that had mutated from BM-MSCs from male rats. Furthermore, we evaluated the tumorigenic and metastatic potential of K3 cells, and we obtained the metastatic K3 cell lines K3-F4 and K3-B6 from liver and lung metastases. Moreover, we characterised the metastatic and cancer stem properties of the K3 cell line and its metastatic cell lines and explored the mechanisms of tumor metastasis.

## RESULTS

### Isolation and identification of the MSC-transformed tumor cell line K3

**Figure 1:**
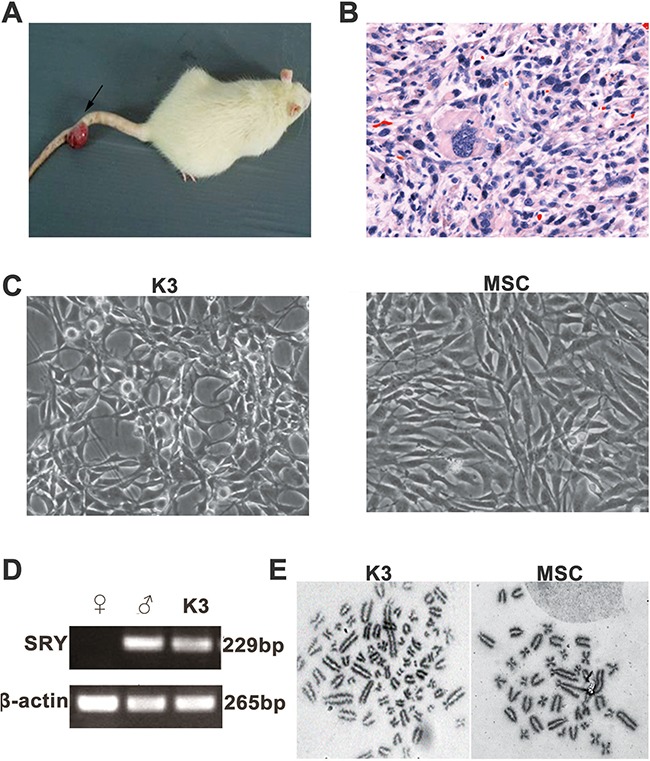
Isolation and identification of the rBM-MSC-transformed tumor cell line K3 **(A)** Tumor on the rat tail (arrow); **(B)** H&E staining of the tumor on the rat tail (400×); **(C)** Morphology of K3 (200×) and rBM-MSC (200×); **(D)** PCR products obtained using the SRY primers: the positive male control (Lane♂) and K3 display the 265-bp β-actin as well as the 229-bp SRY, while the female control (Lane♀) only displays the β-actin product. Lane L contains the 250-bp DNA ladder; **(E)** Karyotype of K3 cell line (with 132 chromosomes) and rat BM-MSCs (with 42 chromosomes).

A novel neoplasm was found on the tail of one female rat 20 months after injection with male rBM-MSCs (Figure 1A). Histopathologic analysis showed the neoplasm to be fibrosarcoma (Figure 1B). The isolated tumor cells exhibited different shapes but gradually became uniform with cell proliferation. We obtained the monoclonal cell line K3 through limited dilution. In contrast to the typical spindle-shaped fibroblast-like morphology of the original rBM-MSCs, the K3 cells showed megagon morphology and appeared smaller (Figure 1C). PCR revealed the existence of the male Sex-determining Region on the Y Chromosome (SRY) in the K3 cells, confirming that K3 cells had transformed from the injected male rBM-MSCs (Figure 1D). Furthermore, we found that the K3 cells exhibited an abnormal hypotetraploid karyotype, with 132 chromosomes, whereas rBM-MSCs exhibited 42 chromosomes as normal (Figure 1E).

### Stemness and tumor characteristics of K3 cells

**Figure 2:**
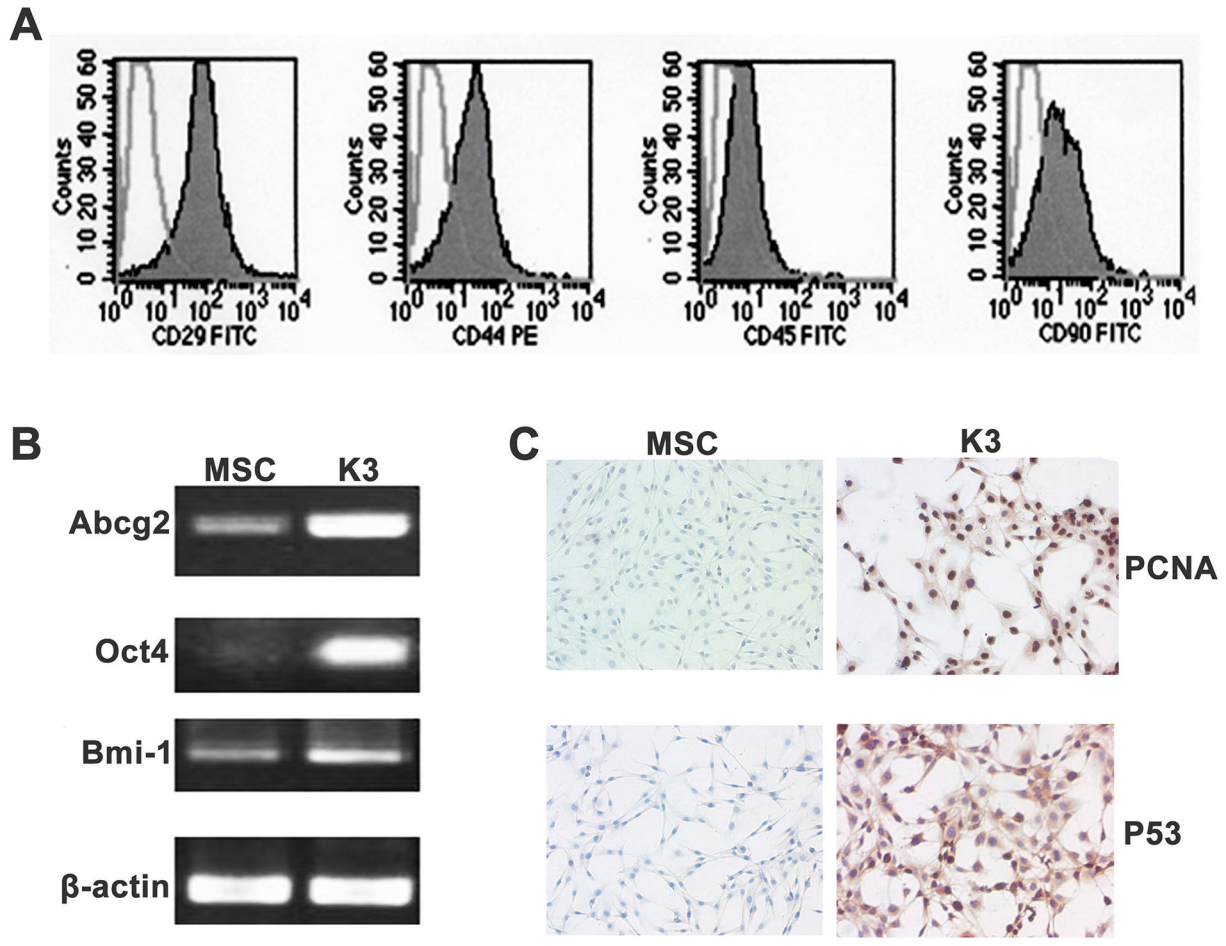
Stemness and tumor characteristics of K3 **(A)** Surface antigens of K3 cells: the cells were positive for CD29, CD44, and CD90 but negative for CD45; **(B)** Analysis of expression of Abcg2, Oct4, and Bmi-1 in MSC and K3 by PCR; **(C)** Immunohistochemistry staining for PCNA and P53 in MSC and K3 (200×).

Comparison of surface markers between the K3 cells and rBM-MSCs revealed that both cells expressed similar surface antigens; they were positive for the rBM-MSC markers CD29, CD44, and CD90, but negative for the hematopoietic marker CD45 (Figure 2A). Moreover, RT-PCR revealed that the K3 cells expressed pluripotent genes such as Abcg2, Oct4, and Bmi-1, and the expression levels were higher than those in rBM-MSCs (Figure 2B). The aforementioned results additionally confirmed that the K3 cells had transformed from the exogenous rBM-MSCs injected *in vivo*. The K3 cells also presented some tumor cell features. Immunohistochemistry analysis showed that the K3 cells were positive for the cell proliferation index (PCNA), and the rBM-MSCs were weakly positive for PCNA (Figure 2C). Similar results were obtained for the tumor-related P53 mutation (Figure 2C). The tumorigenic potential of K3 cells was analysed by generating tumor xenografts through the subcutaneous injection of K3 cells into BALB/c nude mice. All BALB/c nude mice exhibited tumor xenograft formation 20 days after injection with more than 1 × 10^5^ cells. Simultaneously, 1 out of 2 mice exhibited tumor formation after injecting only 1 × 10^4^ cells (Table 1).

**Table 1:** The tumor formation ability of K3 cells in BALB/c mice

No.of injected cells	Days	No.of mice with tumors
10^7^	7	2/2
10^6^	13	1/1
10^5^	20	2/2
10^4^	20	1/2
10^3^	30	0/3

### Characterisation of side population cells (SP) isolated from the K3 cell line

**Figure 3:**
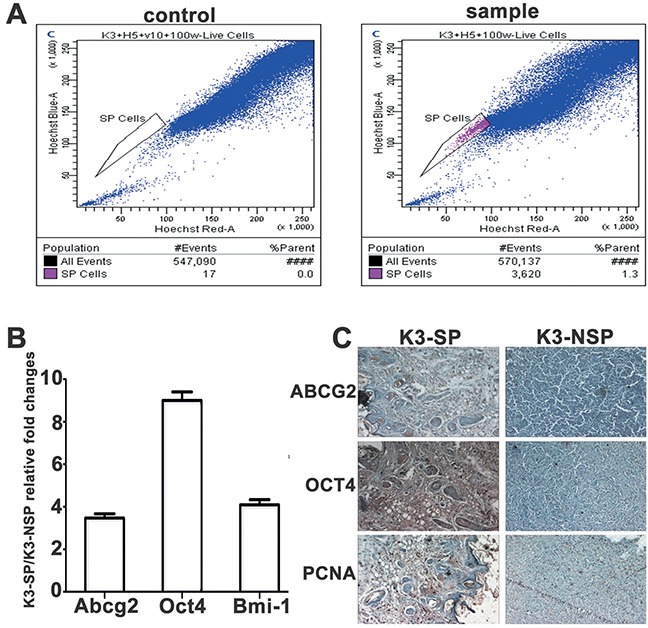
Characterisation of side population cells (SP) isolated from the K3 cell line **(A)** SP phenotype in the K3 cell line: 1.3%; **(B)** qRT-PCR for expression of Abcg2, Oct4, and Bmi-1. Each column represents the fold change in K3-SP/K3-NSP cells (repeated three times); **(C)** Immunohistochemistry staining for ABCG2, OCT4, and PCNA in K3-SP and K3-NSP tumors (100×).

K3 side population (K3-SP) cells were sorted by flow cytometry. The K3 cells were stained blue completely with PI. K3-SP were stained red with 5 μg/ml Hoechst33342 (sample) and were completely inhibited by 50μmol/L verapamil (control). Flow cytometry showed that red dot plot on behalf of the K3-SP cells accounted for approximately 1.3% of the K3 cells (Figure 3A). The remaining non-SP cells were named K3-NSP. The expression levels of stem cell-associated markers Abcg2, Oct4, and Bmi-1 were markedly higher in K3-SP than in K3-NSP cells (Figure 3B). To clarify the differences in the self-renewal ability and tumorigenicity between K3-SP and K3-NSP cells, the two types of cells were subcutaneously injected respectively into the right and left sides of the back of the same BALB/c nude mice. K3-SP exhibited high tumorigenic ability. The injection of only 5,000 K3-SP cells could lead to tumor formation in mice, whereas 20,000 K3-NSP cells were necessary for the same outcome. Furthermore, immunohistochemistry analysis revealed that PCNA, ABCG2, and OCT4 were positive in the tumor tissues caused by K3-SP but negative in the tumor tissues caused by K3-NSP (Figure 3C).

### K3 metastatic cell lines possessed higher metastatic and invasive potential

**Figure 4:**
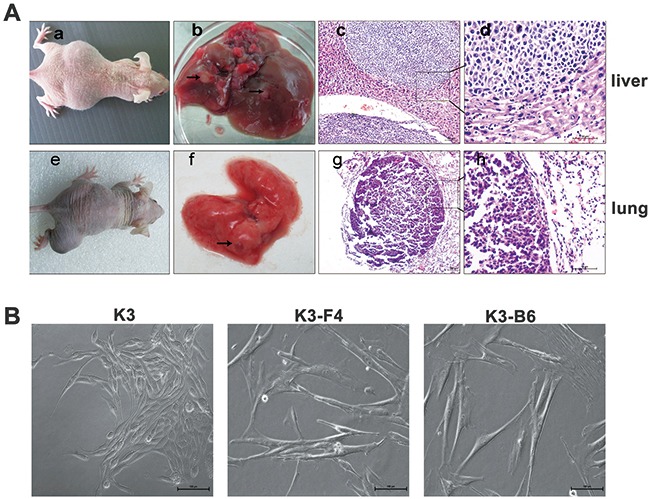
K3 metastatic cell lines possessed higher metastatic and invasive potential **(A)** a. Sign of ascites in mice after their spleen was injected with K3 cells. b. Liver metastasis of K3 (arrows). c, d. HE staining of liver metastasis (×100, ×400); e. Tumor in the right leg where the right proximal tibia was injected with K3 cells. f. Lung metastasis of K3 cells (arrows). g, h. HE staining of lung metastasis (×100, ×400) **(B)** Morphology of primary K3 cell line and liver and lung metastatic cell lines (K3-F4, K3-B6, ×200).

**Figure 5:**
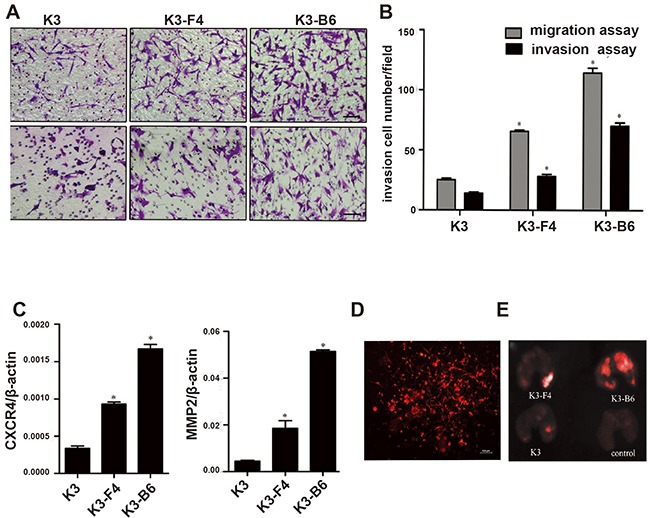
K3 metastatic cell lines induced higher metastatic and invasive potential **(A)** Representative images showing migrating and invading cell morphology (×200); **(B)** *In vitro* migration and invasion assay of K3-F4 and K3-B6 cells. *P < 0.05 (compared with K3 cells, n = 3); **(C)**, mRNA expression of CXCR4 and MMP2 in K3, K3-F4, and K3-B6 cells. *P < 0.05 (compared with K3 cells, n = 3); **(D)** Fluorescence microscopy of stable high-level RFP-expressing K3 cells (×100); **(E)** Images of lung metastatic tumors 30 days after inoculation of K3 cell lines: K3, K3-F4, and K3-B6 cells were injected i.v. at a dose of 1 × 10^5^/100 μl. The control group was injected 100 μl PBS (four animals per group).

Investigating the SP cells was difficult owing to their low proportion in the K3 cells. We established nude mouse metastatic models to study K3 metastatic potential and obtain the cell lines. K3 cells were transplanted into the spleen of BALB/c nude mice, most of which presented with moderate or severe ascites after approximately 30 days (Figure 4A. a). The mice were dissected to detect the condition of their organs, and protuberances were found in the liver (Figure 4A. b), which were confirmed to be tumors by histological analysis (Figure 4A. c, d). Signs of illness (i.e., swelling in the right leg) were observed in the right proximal tibia of BALB/c nude mice after injection with K3 cells (Figure 4A. e). Lumps were observed in the lungs (Figure 4A. f) and subjected to immunohistochemistry analysis (Figure 4A. g, h). Subsequently, we isolated and cultured the metastatic cells and established a liver metastatic cell line named K3-F4 and a lung metastatic cell line named K3-B6. K3-F4 and K3-B6 cells are elongated spindle cells; in contrast to K3 cells, no connection exists between cells in the K3-F4 and K3-B6 cell lines (Figure 4B). We determined whether the isolated variants had increased metastatic potential. Transwell motility and invasion assays showed that the K3-F4 and K3-B6 cell lines were more migratory and invasive than the K3 cell line (Figure 5A, 5B). Compared with K3, the expression of metastasis-related genes (MMP2 and CXCR4) was regulated in the K3-F4 and K3-B6 (Figure 5C). Higher metastatic potential was also confirmed *in vivo*. RFP-labelled K3, K3-F4, and K3-B6 cells were injected into mice through intravenous infusion (Figure 5D). The mice were sacrificed 30 days after transplantation. The Maestro GNIR-Flex CRI *In-vivo* Imaging System was used for fluorescence microimaging. Lung metastasis occurred in all animals except for the control group; regardless of the tumor cell number or the formed tumor area, the metastatic cell lines (K3-F4 and K3-B6) was higher than that of the K3 cell line (Figure 5E).

### K3-F4 and K3-B6 cells exhibited increased tumorigenicity and stemness

**Figure 6:**
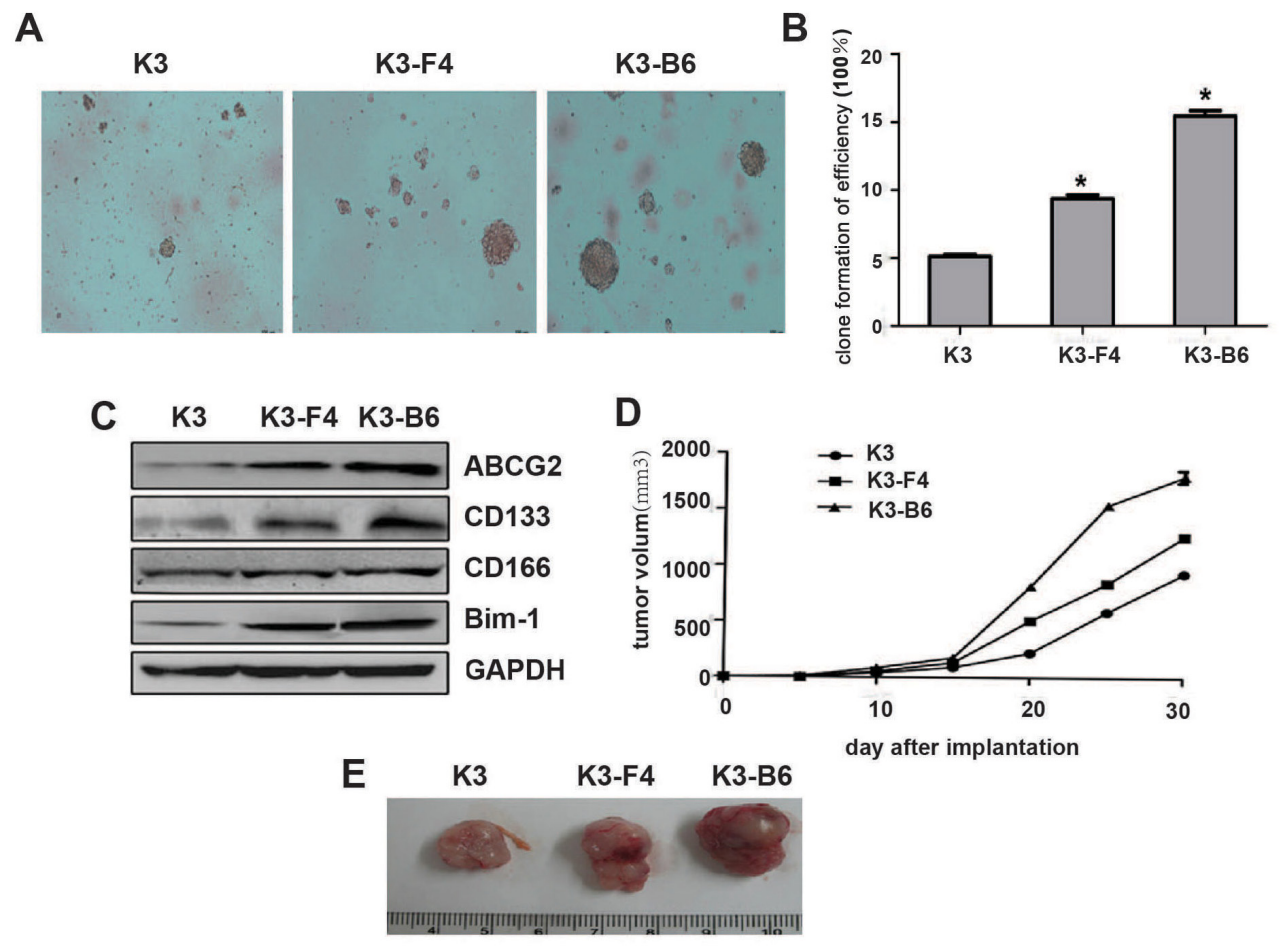
K3-F4 and K3-B6 exhibited increased tumorigenicity and stemness **(A)** Image of clone spheres in soft agar (×100); **(B)** Clone formation efficiency of K3-F4 and K3-B6 cells in comparison with K3 cells. Each column represents the mean of three individual experiments (±SD); *P < 0.005; **(C)** Protein expression level of ABCG2, CD133, CD166, and Bmi-1 in K3, K3-F4, and K3-B6 cells by Western blotting; **(D)** Tumor volume curves; **(E)** K3, K3-F4, and K3-B6 tumor tissue from BALB/c nude mice 30 days after implantation.

A sphere-forming assay revealed that the K3, K3-F4, and K3-B6 cells possessed differentiation ability (Figure 6A), and their colony formation rates were 5.1 ± 0.2%, 9.3 ± 0.4%, and 15.4 ± 0.5%, respectively (Figure 6B). Western blotting also confirmed that the expression of CSC-related surface markers, such as ABCG2, CD133, CD166, and Bmi-1, was increased in K3-F4 and K3-B6 cells compare with in K3 cells, with maximum expression in K3-B6 cells (Figure 6C). In addition, nude mice transplantation showed that tumors caused by K3-F4 and K3-B6 cells grew more rapidly compared with those caused by K3 cells (Figure 6D, 6E).

### EMT induced the transformation of K3 cells into K3-F4 and K3-B6 cells by upregulating the stemness and metastatic capacity of K3 cells

**Figure 7:**
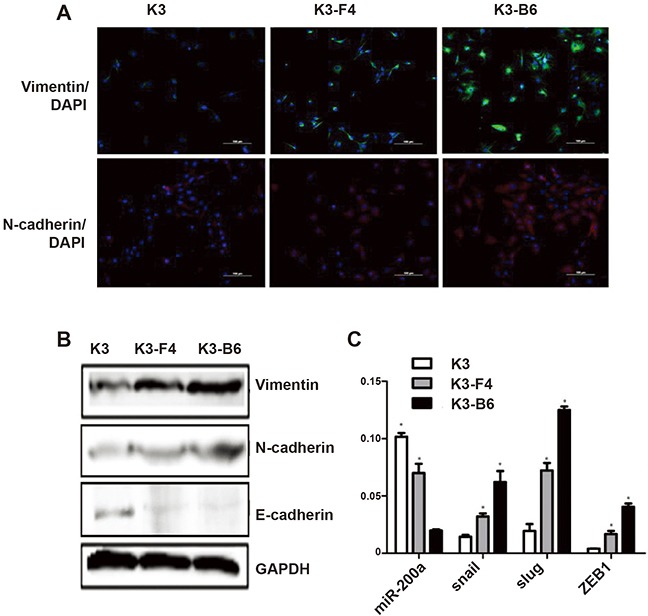
EMT induced transformation of K3 into K3-F4 and K3-B6 by upregulating the stemness and metastatic capacity of K3 **(A)** Expression of vimentin and N-cadherin in K3, K3-F4, and K3-B6 cells was detected by immunofluorescence analysis (×200); **(B)** Expression of vimentin, N-cadherin, and E-cadherin in K3, K3-F4, K3-B6 cells was detected by Western blotting; **(C)** Expression of miRNA-200a, snail, slug, and ZEB1 in K3, K3-F4, and K3-B6 cells was detected. *P < 0.05 (compared with K3 cells, n = 3).

The morphology of K3 cells differs from those of its metastatic cell lines K3-F4 and K3-B6. K3 cells are small and spindle-shaped (Figure 1C), and K3-F4 and K3-B6 cells are long and spindle-shaped with more pseudopods (Figure 4B). We examined some EMT-related genes to explain the mechanisms of morphological changes and motility among these cells. Immunofluorescence analysis revealed that the expression levels of the mesenchymal markers vimentin and N-cadherin were higher in K3-F4 and K3-B6 cells than in K3 cells (Figure 7A). Western blotting showed the same results and confirmed that the expression of the epithelial marker E-cadherin had decreased in K3-F4 and K3-B6 cells (Figure 7B). Moreover, the expression levels of miR-200a, an accepted EMT inhibitor, were lower in K3-F4 and K3-B6 cells than in K3 cells (Figure 7C). RT-PCR also showed that the expression of EMT-related transcription factors such as snail, slug, and ZEB1 was higher in K3-F4 and K3-B6 cells than in K3 cells (Figure 7C).

## DISCUSSION

Mesenchymal stem cells (MSCs) have self-renewal and multilineage properties. BM-MSC can home and differentiate into adult cells. Many researches have showed human BM-MSCs transplantation treated disease [1]. However, MSCs have been found to participate in the tumor microenvironment [8] and promote tumor progression [9, 10]. Therefore, it is important to study the safety of mesenchymal stem cells. We first established a novel tumor cell line named F6 that was mutated from human embryonic BM-MSCs *in vitro* [6]. In this study, a novel neoplasm was found on the tail of female rat after injection with male rBM-MSCs. We isolated the new tumor cell line named K3. Purification of K3 is the most important process in cell isolation and culture. The colonies of fibroblast-like cells appeared after cell migrating from the tumor tissues and adhering to the plastic surface. Cells were cloned in the 96-well plate after six passages using the method of limited dilution, by which cells were diluted till only one a well. Finally, this cell clone proliferates to form K3. The existence of the male Sex-determining Region on the Y Chromosome (SRY) in the K3 cells, confirming that K3 cells had transformed from the injected male rBM-MSCs. K3 cells express surface antigens similar to BM-MSCs and possess tumorigenicity and stemness. Moreover, K3 cells exhibit high metastatic potential and we construct two cell lines, K3-F4 and K3-B6, from liver and lung metastatic tumor tissues.

Recently, studies have confirmed that CSCs corelate with the initiation of tumors [11, 12] and the progression of cancers such as acute leukaemia [13], breast cancer [14], prostate cancer [15], and ovarian cancer [16]. Some investigators suggest that CSCs are derived from mesenchymal cells, including MSCs [17]. In our previous study, F6 cells transformed from human MSCs contained a small population of CSCs that contribute to its heterogeneity and tumorigenic potential [7]. This study showed that transformed MSCs *in vivo* also contained a small population of CSCs. Therefore, we speculate that mutated MSCs are potential origin of CSCs. Differences were observed in tumorigenic capacity and stemness-related gene expression between K3-SP and K3-NSP cells. K3-SP cells exhibited higher tumorigenic capacity than did K3-NSP cells. On the basis of the aforementioned results, we hypothesized that a small fraction of MSC was transformed into K3-SP, and then these K3-SP differentiated into tumor cells that were K3. As to the authenticity of this hypothesis, it is necessary to study and confirm.

In our study, K3-F4 and K3-B6 cells exhibited higher metastatic potential than K3 cells, which was confirmed by *in vitro* and *in vivo* experiments. EMT promotes the progression of tumorigenicity [18–20] and mediates the stem cell phenotype [21, 22]. EMT may be the reason for K3 transforming to K3-B6, K3-F4. We found that the expression of the mesenchymal markers vimentin and N-cadherin and EMT inducers was increased in K3-F4 and K3-B6 cells compare with in K3 cells. In addition, the expression level of the accepted EMT inhibitor miR-200a was lower in K3-F4 and K3-B6. In summary, EMT might cause the malignant transformation of K3 cells and be responsible for enhancing metastatic potential and cancer stemness.

In conclusion, this study is the first to prove the existence of mutated MSCs *in vivo*, and successfully established the transformed cell line K3. The characteristics of K3 and its metastatic cell lines K3-F4 and K3-B6 were investigated. EMT is a potential mechanism of K3-F4 and K3-B6 cell formation. Our study provides new evidence to investigate the safety of the clinical application of MSCs and new insights into the mechanism of tumorigenesis, which may be of crucial relevance to the realms of theory and application.

## MATERIALS AND METHODS

### The original of novel mutated rBM-MSC tumor

Rat bone marrow mesenchymal stem cell (rBM-MSCs) were isolated from male Wistar rats. The cells in the femoral bone were flushed out with phosphate-buffered saline (PBS), collected by centrifugation at 800 g for 5 min, and cultured in Dulbecco's modified Eagle's medium with low glucose (LG-DMEM, Gibco, USA) containing 10% fetal bovine serum (FBS, Gibco, USA). The medium was changed at 72 h after cells adhered to the culture dishes. When 80% confluence was reached, the cells were trypsinized with 0.25% trypsin-EDTA (Amresco, USA) and passed into new dishes for further expansion. Then the cells were identified as mesenchymal stem cells by surface marker identification. The male rat bone marrow MSCs (2×10^6^) were intra-arterially injected into a female rat tail for experiments at day 0, 3, 7, 14, 21, we found that two of ten rats formed a foreign body in tails after 20 months and stripping out these bodies to process Hematoxylin–eosin (H&E) assay. Excitingly, it was considered as a novel neoplasm.

### Isolation and culture of K3 cells

Fresh tumor xenografts were harvested and then washed by PBS with antibiotics several times to remove the blood. After removing the surface of the tumor tissues, the inner parts were cut into 1-3 mm^3^ sized pieces and floated in LG-DMEM containing 10% FBS, penicillin (100 U/ml) and streptomycin (100μg/ml). Subsequently, the pieces of cancer tissues were incubated at 37°C in humidified air with 5% CO_2_ for 3 days, cells had migrated from the tumor tissues and adhered to the plastic surface and colonies of fibroblast-like cells appeared. The cell line was denominated as K3 cells, cloned in the 96-well plate after six passages using the method of limited dilution, by which cells were diluted till only one a well. The cell line was denominated as K3.

### Flow cytometry

The K3 cells were immunostained with monoclonal antibodies, such as fluorescein isothiocyanate FITC-CD29, CD90, CD45, and PE-CD44. The antibodies FITC-IgG1 and PE-IgG1 were used in the control group. The cells were analysed using a flow cytometer.

### Side population cells (SP) sorting and culture

The cells were detached from the dishes with 0.05% trypsin EDTA and resuspended at 2×10^6^ cells/ml in DMEM with 2% FBS. The cell suspensions were incubated with 5 μg/ml Hoechst33342 (Sigma, USA) for 90 min at 37°C either alone or in the presence of 50μmol/L verapamil (Sigma, USA) at 37°C for 30 min. The cells were resuspended in PBS supplemented with 2% FBS. Cell analysis and sorting were performed using FACS Aria II SORP (BD Biosciences, USA). Cells were displayed on dot plots, PI negative, and viewed in a Hoechst Blue versus Hoechst Red dot plot to visualize the SP cells.

### RNA and DNA extraction and reverse transcription polymerase chain reaction

Total RNA was extracted using Trizol reagent (Invitrogen, USA). DNA was extracted using the QIAamp DNA Mini Kit (Qiagen, USA). Reverse transcription polymerase chain reactions (RT-PCR) were performed with the RevertAid First Strand cDNA Synthesis Kit (Fermentas, Glen Burnie, MD, USA) and the BioEasy SYBR Green I RT-PCR Kit (Bioer Technology, Hangzhou, China). Table 2 lists the primers designed to generate specific products.

**Table 2:** Primer sequences for the amplication of target genes and β-actin

Genes	Primer sequences(5′-3′)	Amplicon size (bp)	Annealing temp (°C)
SRY	F:CCAGCATGCAGAATTCAGAG	229	59
	R:CATTGCAGCAGGTTGTACAG		
β-actin	F:CACGAAACTACCTTCAACTCC	265	56
	R:CATACTCCTGCTTGCTGATC		
Abcg2	F:TGTCAGCTGTGGAGCTCTTC	244	60
	R:ATGGAACTGGCCGTATAAGC		
Oct-04	F:TGCAGCTCAGCCTTAAGAAC	265	59
	R:AACCACACTCGAACCACATC		
Bmi-1	F:CGTCGGAACAAGAGACTCTG	242	59
	R:AGGCTGACATCTGCTCTCAC		
CXCR4	F:ACGGCAGGTACATCTGTGAC	296	59
	R:GACGCTCTCGAACTCACATC		
MMP2	F:AGATTGATGCCGTGTACGAG	273	59
	R:AGGAGTCTGCGATGAGCTTC		
Slug	F:GTCCGAATGTGCATCTTCAG	276	59
	R:CCTGCCTTCATCTGACACTT		
Snail	F:ACTATAGCGAGCTGCAGGAC	233	58
	R:GTCCTCATCGGACAGAGAAG		
ZEB1	F:GAGCACTTACGGATCCACAG	246	60
	R:AACAACAGCTTGCACCACAC		
miR-200a	RT:GTCGTATCCAGTGCAGGGTCCGAGGTATTCGCACTGGATACGACACATCG	66	60
	F:GCCGCTAACACTGTCTGGTA		
	R:GTGCAGGGTCCGAGGT		

### Immunohistochemistry

Tumor tissue sections were first incubated with antibodies against ABCG2, OCT4, P53, and the proliferating cell nuclear antigen (PCNA), according to manufacturer instructions (eBioscience, USA). Subsequently, the sections were incubated with the secondary antibody and reacted using 3,3′-diaminobenzidine reagent. Finally, the sections were mounted with neutral gum for microscopic examination.

### Nude mouse model of K3 cell metastasis and metastatic cell lines

The K3 cells were injected into the spleen or the right proximal tibia of female BALB/c nude mice. After the development of lung and liver tumors originating from migrating K3, the metastatic K3 cell lines correspondingly named K3-B6 and K3-F4 were isolated from these tumors.

### Tumorigenicity assay *in vivo*

BALB/c nude mice were purchased from the Shanghai Laboratory Animal Center, Academy of Sciences, China. K3, K3-F4, and K3-B6 cells were injected subcutaneously into BALB/c nude mice. The tumor size was monitored using calliper measurements.

### Western blotting

Proteins were separated on a 12% sodium dodecyl sulfate polyacrylamide gel electrophoresis gel and then transferred onto polyvinylidene fluoride membranes. The membranes were incubated with primary antibodies (ABCG2, CD133, CD166, Bmi-1, vimentin, N-cadherin, E-cadherin, GAPDH, 1:300, SAB, USA), followed by incubation with horseradish peroxidase-conjugated goat anti-rabbit antibody (1:2000). The antibody-antigen complexes were visualised using the ImageQuant LAS 4000 mini (GE Company, USA).

### Soft agar cloning assay

A total of 5 × 10^4^ cells were suspended in 10 ml of 0.3% agar (Sigma, USA) supplemented with the culture medium. Thereafter, 1 ml of the cell suspension per well was layered over the bottom layer of 0.6% agar in 6-well plates. Culturing was continued until colonies of > 50 cells were obtained.

### Cell transfection and fluorescence imaging

The recombinant vectors (pcDNA3.0-mLumin) containing mLumin and a neomycin resistance gene (neo) were transfected into K3, K3-F4, and K3-B6 cells using the calcium phosphate method. The red fluorescent protein (RFP)-positive colonies were selected by G418 (Sigma, USA) and were injected into mice using intravenous infusion. The Maestro GNIR-Flex CRI *In-vivo* Imaging System was used for fluorescence microimaging.

### Immunofluorescence

Cells were fixed in 4% paraformaldehyde and incubated with a primary antibody (vimentin, N-cadherin, 1:50, SAB, USA), followed by incubation with a FITC- or CY3-conjugated secondary antibody. The nuclei were counterstained with DAPI and analysed under a confocal microscope (TCS SP5, Leica, Germany).
